# EBV Chronic Infections

**DOI:** 10.4084/MJHID.2010.022

**Published:** 2010-08-10

**Authors:** Pizzigallo Eligio, Racciatti Delia, Gorgoretti Valeria

**Affiliations:** Clinic of Infectious Diseases – Department of Medicine and Aging – “G. D’Annunzio” University of Chieti (Italy)

## Abstract

The infection from Epstein-Barr virus (EBV) or virus of infectious mononucleosis, together with other herpes viruses’ infections, represents a prototype of persistent viral infections characterized by the property of the latency. Although the reactivations of the latent infection are associated with the resumption of the viral replication and eventually with the “shedding”, it is still not clear if this virus can determine chronic infectious diseases, more or less evolutive. These diseases could include some pathological conditions actually defined as “idiopathic”and characterized by the “viral persistence” as the more credible pathogenetic factor. Among the so-called idiopathic syndromes, the “chronic fatigue syndrome” (CFS) aroused a great interest around the eighties of the last century when, just for its relationship with EBV, it was called “chronic mononucleosis” or “chronic EBV infection”.

Today CFS, as defined in 1994 by the CDC of Atlanta (USA), really represents a multifactorial syndrome characterized by a chronic course, where reactivation and remission phases alternate, and by a good prognosis. The etiopathogenetic role of EBV is demonstrated only in a well-examined subgroup of patients, while in most of the remaining cases this role should be played by other infectious agents - able to remain in a latent or persistent way in the host – or even by not infectious agents (toxic, neuroendocrine, methabolic, etc.). However, the pathogenetic substrate of the different etiologic forms seems to be the same, much probably represented by the oxidative damage due to the release of pro-inflammatory cytokines as a response to the triggering event (infectious or not infectious).

Anyway, recently the scientists turned their’s attention to the genetic predisposition of the subjects affected by the syndrome, so that in the last years the genetic studies, together with those of molecular biology, received a great impulse. Thanks to both these studies it was possibile to confirm the etiologic links between the syndrome and EBV or other herpesviruses or other persistent infectious agents.

The mechanisms of EBV latency have been carefully examined both because they represent the virus strategy to elude the response of the immune system of the host, and because they are correlated with those oncologic conditions associated to the viral persistence, particularly lymphomas and lymphoproliferative disorders. Just these malignancies, for which a pathogenetic role of EBV is clearly documented, should represent the main clinical expression of a first group of chronic EBV infections characterized by a natural history where the neoplastic event aroused from the viral persistence in the resting B cells for all the life, from the genetic predisposition of the host and from the oncogenic potentialities of the virus that chronically persists and incurs reactivations.

Really, these oncological diseases should be considered more complications than chronic forms of the illness, as well as other malignancies for which a viral – or even infectious - etiology is well recognized. The chronic diseases, in fact, should be linked in a pathogenetic and temporal way to the acute infection, from whom start the natural history of the following disease. So, as for the chronic liver diseases from HBV and HCV, it was conied the acronym of CAEBV (Chronic Active EBV infection), distinguishing within these pathologies the more severe forms (SCAEBV) mostly reported in Far East and among children or adolescents. Probably only these forms have to be considered expressions of a chronic EBV infection “sensu scrictu”, together with those forms of CFS where the etiopathogenetic and temporal link with the acute EBV infection is well documented. As for CFS, also for CAEBV the criteria for a case definition were defined, even on the basis of serological and virological findings. However, the lymphoproliferative disorders are excluded from these forms and mantain their nosographic (e.g. T or B cell or NK type lymphomas) and pathogenetic collocation, even when they occur within chronic forms of EBV infection. In the pathogenesis, near to the programs of latency of the virus, the genetic and environmental factors, independent from the real natural history of EBV infection, play a crucial role.

Finally, it was realized a review of cases - not much numerous in literature – of chronic EBV infection associated to chronic liver and neurological diseases, where the modern techniques of molecular biology should be useful to obtain a more exact etiologic definition, not always possibile to reach in the past.

The wide variety of clinical forms associated to the EBV chronic infection makes difficult the finding of a univocal pathogenetic link. There is no doubt, however, that a careful examination of the different clinical forms described in this review should be useful to open new horizons to the study of the persistent viral infections and the still not well cleared pathologies that they can induce in the human host.

## Introduction:

*“Once infected, always infected”.* Introduced in order to express in synthetic way the pathogenetic essence of the infection from *Cytomegalovirus*, whose natural history constitutes one of the more classic examples of persistence of an infectious agent in the human organism, this maxim can be applied to an always increasing number of infections, both viral and not. The concept that it synthetizes represents one of the more interesting and innovative aspects of the modern infectivology. This one is more and more very rarely represented from acute infectious diseases that can effectively be resolved with anti-infectives drugs and by now be prevented with vaccines. The persistent and/or chronic infections - in which the microorganism does not come eliminated from the host but persists for months, years or even all the life - emerge instead, and they end with prevailing. This happens for the conditions of immunodeficiency of the host whose immunity answer is not able to eliminate the infectious agent, even if can partially slow down the pathogenic action. Furthermore, it also depends from the biological characteristics of the virus that determines its persistence (through the latency or the fast mutation towards less virulent forms able to persist more over a long time or even indefinitely in the host, without necessarily cause a damage).

In the within of the persistent viral infections it is important to distinguish those in which there are an active replication and release of the virus for a long period of time and where the virus can be often cultivated or identified (e.g.: through the techniques of molecular biology), that we can properly indicate as *chronic viral infections*. There are then the *latent viral infections* in which the virus persists in a form unable to be identified through standard methods as the coltural exams or electronic microscopy; neither the latency can be revealed from the immune system of the host. At worst this last one can evidence the “reactivation” of the latent infection, when it occurs. It involves the production of new virions and eventually the “shedding” of complete viral particles. This means that throughout the latency the viral genome must be present all complete: the more important mechanism through which this happens, but sure not the only, is the integration of the viral genome in that one of the cell host (e.g.: infection from hepatitis B virus or HBV).^1^,^2^,^3^,^4^

Among the persistent viral infections characterized from the latency those supported from the family of *Herpesviridae* are above all. In this kind of infections the reactivation can be accompanied to a resumption of the viral replication, and eventually to the “shedding”, but it is discussed if it can induce chronic infectious diseases, more or less evolutive.

This is particularly true for the *Epstein-Barr virus* (EBV) or virus of the *infectious mononucleosis* (IM), sure endowed, like all the herpetic viruses, of the property of latency and like such it is sure implied in the pathogenesis of several onco-haematological diseases. It is less clear instead if forms of EBV chronic infection able to induce evolutive pathologies can exist, like for the hepatitis B or C, through eventually the more or less frequent reactivation of the latent infection.

The search of possible chronic forms of EBV infection must not consider only eventual persistent mononucleosis syndromes that of it would represent the natural clinico-pathogenetical equivalent as well as the lymphoproliferative forms for which the correlation with the virus at this point is definitively established. It is necessary in fact to address to some pathological conditions currently defined like “idiopathic” for which the phenomenon of the “viral persistence” seems to represent the more reliable pathogenetic element.^5^

Between these pathologies we could mention diseases like rheumatoid arthritis, systemic erythematous lupus, multiple sclerosis and above all the “chronic fatigue syndrome” in which a role of EBV still remains controversial and of difficult demonstration, even though many Authors hypothesized a direct or indirect role of such virus.^6^

## EBV and diseases

### Virologic features and acute or persistent infection:

Epstein-Barr virus (EBV) belongs to *Herpesviridae* family, which includes various viruses that have co-evoluted with their respective hosts over millions of years and during this time have developed sophisticated strategies for lifelong persistence that are beneficial for their survival and spread.^7^

The structure of EBV shows a linear double-stranded DNA, an icosadeltahedral capside, 162 capsomers and an envelope. The EBV genoma encodes nearly 100 viral proteins.^8^ During viral replication, these proteins play a fundamental role in regulating the expression of viral genes, replicating viral DNA, forming structural components of the virion, and modulating the host immune response.

The receptor for the virus on epithelial cells and B lymphocytes is the CD21 molecule, formerly called CR2, which is also the receptor for the C3d component of complement.^9^ *In vitro* the infection of B lymphocytes by EBV determines a continuous cell proliferation, which results in a cellular immortalisation; the infection of epithelial cells, instead, results in an active replication, with production of virions and host cell lysis.^10^ *In vivo* there are other factors in addition to CD21 that are important to determine the infection. The major histocompatibility complex (MHC) class II molecule serves as cofactor for the infection of B cells.^11^

Cellular infection from EBV could have two possible outcomes. A lytic infection occurs wherein virions are produced and the host cell is lysed. Lytic infection tipically occurs in epithelial cells and partly in plasma cells.^12^ Alternatively, EBV may induce a latent infection by generating an episome, the circular EBV genoma, that is located in the nucleus of host lymphocytes. This episome remains in a latent form in B cells;^13^ viral replication is spontaneously activated in only a small percentage of latently infected B cells.

Infection of humans from EBV usually follows the contact with oral secretions. The viral replication occurs in cells of the oropharynx, and nearly all seropositive persons actively shed virus in the saliva. Although earlier studies indicated that the virus replicated in epithelial cells of the oropharynx,^10^ and investigators postulated that B cells were subsequently infected after contact with these cells, other studies suggest that B cells in oropharynx may be the primary site of infection.^14^

*Primary infection* in young children usually occurs in an asymptomatic way but, in 50% of adolescents, it causes infectious mononucleosis (IM). In healthy individuals, the primary infection comes to a resolution and the virus establishes a harmless latent, persistent infection.

IM usually presents with the acute onset of fever, sore throat and enlarged and painful lymph glands in the neck. However, the more typical clinical feature of the disease, in a position to distinguish it from other viral and bacterial throat infections, is represented by a severe and debilitating fatigue which accompanies these symptoms and which may last for months after they have resolved.

Primary EBV infection induces both humoral and cellular immune response that control but do not eliminate the infection. Antibodies are generated to both latent and lytic antigens, and those directed against the virus receptor on the viral envelope, glycoprotein 350 (gp350), prevent binding to CR2 on B cells and thereby limit viral spread and prevent reinfection.^7^

Immunoglobulin M antibodies to the EBV viral capside antigen (anti-VCA) evolve quickly with infection, persist for weeks to months, and do not reappear. Thus, their detection is presumptive evidence of recent primary infection. IgG anti-VCA shows a rise, a subsequent fall, and a lifelong persistence usually in titers ranging from 1:40 to 1:2560. Antibodies to viral early (pre-DNA synthesis) antigens (anti-EA) of the diffuse (D) or restricted (R) types develop in most primary infections, peak in titers of less than 1:640, and wane with time. Antibody to EBV nuclear antigen (anti-EBNA) are detected in traditional assay relatively late after the onset of symptoms in IM, so that their absence in previously well person who develops acute illness suggest an ongoing EBV infection.^15^

The response of CD8-positive (CD8+) cytotoxic T-cells is crucial to control the primary infection, and these cells show a predominant role in IM, being present in the circulation and tissues in very high numbers. These cells probably give rise to most of the symptoms and signs of IM as a result of massive production of cytokines, including lymphotoxin, tumor necrosis factor-α, interleukin (IL)-1β and IL-6.^16^

That CD8+ T cells are essential for recovery from IM is exemplified by the consequence of primary EBV infection in immunocompromised individuals who are unable to mount the appropriate response and who usually die of a fulminating IM-like syndrome within weeks of acquiring EBV. This is the case of childrens affected by the X-linked lymphoproliferative syndrome (XLPS), where some of them, survived to the initial infection, develop in a second time a B-cell lymphoma and/or dysgammaglobulinemia.

As previously reported, after the primary infection the virus persists in the latent episomal form in the nucleus of B lymphocytes and B-cell-resting.^13^

Resting memory B cells represent the site of *persistence* of EBV within the body.^17^ In normal adults, from 1 to 50 B cells per million in the circulation are infected with EBV, and the number of latently infected cells within a person remains stable over years.

Of the nearly 100 viral genes that are expressed during replication, only 10 are expressed in latently infected B cells *in vitro*: two types of nontranslated RNA, six nuclear protein and two membrane proteins.

By markedly limiting viral gene expression during latency, EBV reduces the number of viral proteins that permit the recognition of infected cells by cytotoxic T cells. Our knowledge of the functions of the latent proteins is extensive but incomplete (**Table 1**): the main functions so far explored are represented by the maintenance of latency, the immortalization of B cells *in vitro* and the oncogenetic activity.

Analysing EBV gene expression in B-cells subsets from peripheral blood and tonsil, Thorley-Lawson and Babcock^18^ suggest that latent infections can be further subdivided into four infection programs (**Table 2**). In the first form, only EBNA-1 and EBER are expressed, whereas in the second form EBNA-1, LMP-1, LMP-2, and EBER are expressed. In the third pattern, all the latency genes are expressed. A fourth pattern of latency is seen in B cells obtained from the peripheral blood of healthy persons infected with EBV in the past, in which only EBER and LMP-2, and in some studies, EBNA-1 RNA have been detected.^19^

### Malignancies associated with latent and/or persistent infection by EBV:

The different programs of EBV latency, whose existence should be the result of a deliberate strategy of the virus to survive to the immunological response of the host, have allowed to correlate some of them with malignancies typically associated with viral persistence (**Table 2**).

In these pathological conditions the latency of EBV is often accompanied by low level, continuous or intermittent production of infectious virions into the saliva.^20^ So Burkitt’s lymphoma should be characterized by the type 1 of the latency program, the nasopharyngeal carcinoma, Hodgkin’s lymphoma and peripheral T cell lymphoma by the type 2. The type 3 of the latency program should characterize XLPS and other lymphoproliferative diseases induced by EBV in immunocompromised hosts (bone marrow and solid organ transplanted, etc.), as well as the IM (**Table 2**).^13^

We will not go into the merits of the pathogenic mechanisms by which latent EBV infection induces lymphoproliferative disease and other malignancies with which it was related, even because this argument was recently treated in others reviews of this journal.^21^,^22^,^23^,^24^,^25^ However, it is well established that these mechanisms involve virological factors associated with EBV antigenic characteristics, host factors with particular regard to the genetic and immune systems and environmental co-factors (see for example the malaria in Burkitt’s lymphoma and the consumption of “salade fish” in nasopharyngeal carcinoma). There is no doubt, moreover, that precisely these malignancies, for which the pathogenetic role of EBV has been conclusively shown, may represent the clinical expression of a first group of EBV chronic infections in which the natural history sees the neoplastic event, albeit conditioned by numerous other factors, not only as the result of the persistence of EBV in resting B-cells throughout life (so the host is exposed to potentially oncogenic viral gene products for a long period of time), but also as the result of the virus ability to encode grown promoting genes, such as EBNA-2 and LMP-1, and of the reactivation of a latent infection.^6^

## Chronic EBV Infection “*sensu strictu*”

The term “chronic EBV infection” should be really referred to the possible chronic evolution of clinical pictures associated to primary EBV infection, symptomatic or asymptomatic, while the EBV-associated neoplastic diseases, at least momentarily, must be excluded. The clinical evolution of these forms over time would determine the discharge of the classical features of the EBV infection and the assumption of the typical features of real complications.

Although symptoms associated with the EBV acute illness (IM) typically resolve in the first month, a prolonged recovery period associated with clinical and laboratory sequelae has been reported in the medical literature.^26^,^27^,^28^ An early description of “chronic mononucleosis syndrome” cited “weakness, aching legs, low-grade fever, and depression” as typical symptoms.^29^

However, it was only in the mid 80s of last century that the problem of “chronic mononucleosis” or chronic EBV infection has captured the attention of researchers, following a series of clinical studies that have described cohorts of patients with prolonged atipical illness with persistent fatigue, headaches, myalgia, lymphadenopathy, and intermittent and/or low-grade fever.^30^,^31^,^32^,^33^,^34^,^35^,^36^ Unexpectedly, unusual profiles of antibodies to EBV were common in this syndrome. Significantly, the titers of EBV IgG antibodies to VCA and EA were substantially higher in patients than in controls.

Because of these serologic patterns and clinical symptoms, it was proposed that the syndrome arose from a chronic EBV infection.^37^

Hellman et al.^38^ first proposed the acronym CAEBV (= Chronic Active EBV infection) for this enigmatic disease that, however, seemed to have a good prognosis despite some reports, of Virelizier et al.^39^ first and Joncas et al.^40^ later, about forms of CAEBV characterized by more severe and often fatal clinical manifestations (SCAEBV). Since the first reports of this new emerging syndrome potentially related to EBV, the NIH in Bethesda (USA) took on a task to coordinate a study group that in April 1985 reported the preliminary findigs^33^,^34^ in a workshop during which the investigators divided the examined patients into 3 groups, mainly by EBV serologic results. The first group consisted of rare persons with specific lymphoproliferative, hypoplastic or other disorders, with some degree of demonstrable immune impairment and extraordinarily elevated titers of antibodies to EBV-related antigens: IgG antibody titers to VCA of ≥ 5,120 and to EA diffuse [D] or restricted [R] of ≥ 640, and with low or absent antibody titers to EBV-determined nuclear antigen (EBNA). The second group included a much larger number of patients with chronic fatigue, with no obvious immunodeficiency, and with variable but less impressive or normal EBV antibody titers. This group was further subdivided as to whether the patients had experienced an episode of acute IM. The third, small group of patients included those who could not be clinically distinguished from others with chronic fatigue but who lacked all antibodies to EBV.

At this point it became clear that clinical studies about this mysterious syndrome, characterized by a severe asthenia and marked fatiguability, as well as by many other clinical symptoms well explained by a persistent EBV infection, have begun to divide, so that a division of patients into different groups,^41^ not all necessarily linked to chronic EBV infection alone (**Table 3**), was proposed, and namely:

1. Chronic Fatigue Syndrome of unknown etiology (CFS)
2. Chronic Active EBV infection (CAEBV)
3. Severe Chronic Active EBV infection (SCAEBV).

We will return later on CFS, which is mainly characterized by debilitating fatigue, so to discuss the possible pathogenetic role of EBV, since this syndrome seems a multifactorial disease with a pathogenesis still not well defined.

Therefore, at the moment we will take into consideration only the forms of chronic EBV infection in which the etiopathogenetic relationship with the virus is demonstrated by reliable virological and/or immunological studies. Probably, the meaning of illness caused by chronic active EBV infection “sensu strictu” can be correctly attributed only to these clinical forms.

### a) Chronic Active EBV infection (CAEBV):

An high titer of anti-VCA antibodies for EBV, the frequent presence of IgA and IgM anti-VCA with persistently high levels of anti-EA (D) and/or EA (R), and occasionally the positivity of IgA anti-EA (D) represent some of the major viro-immunological characteristics of patients affected by CAEBV. Some patients also lack anti-EBNA antibodies which usually represent a specific immune response to EBV infection (**Table 3**). More recently the identification of EBV-DNA in peripheral blood mononuclear cells (PBMCs) and tissues show an higher diagnostic significance.^42^,^43^,^44^,^45^

The largest group of patients characterized by chronic or recurrent infectious mononucleosis-like symptoms persisting over a long time and by unusual pattern of anti-EBV antibodies^37^ is probably represented by forms of CAEBV CFS-like. Patients with this syndrome do not show any prior immunologic abnormalities or any other sign of a recent infection that might explain their condition.^36^

There are then the more severe forms of CAEBV described mainly but not exclusively in Japan.^46^,^42^,^47^,^41^,^48^,^49^,^50^ They are often characterized by an unfavourable prognosis because of hematologic, gastroenteric, neurological, pulmonary, ocular, dermatological and/or cardiovascular complications.

The more interesting cardiovascular disorders of patients with CAEBV are represented by aneurysms of the coronaries and valve abnormalities.^51^ Moreover, in the course of the disease, lymphoproliferative disorders like hemophagocytic lymphohistiocytosis, lymphoproliferative diseases and T or NK cell lymphomas or various kind of cutaneous manifestations (including hydroa vacciniforme), in addition to hypersensitivity to insect bites, may occur.

The large number of studies by Japanese authors^52^,^53^,^54^ has identified criteria to highlight the differences that undoubtedly exist between such clinically heterogeneous patients, where the only link seems to be the correlation with chronic EBV infection, as demonstrated by the previously reported peculiar sero-immunological aspects.

The three main criteria of CAEBV infection were established by Straus^36^ and subsequently Okano et al.^55^,^52^ have proposed similar criteria even for the most severe form of CAEBV (**Table 4 and 5**). Importantly, there are many cases that do not satisfy the criteria described above.

Some patients lack abnormal patterns of EBV-related antibodies, whereas other patients lack major organ involvement or show only skin symptoms, such as hypersensitivity to mosquito bites 56,57.

On the other hand, Kimura et al.^43^ and Maeda et al.^44^ have reported in patients with CAEBV infection extremely high viral loads, as assessed by quantitative polymerase chain reaction (PCR). Furthermore, the disease severity directly correlates with levels of EBV-DNA in serum or plasma.^58^ In addition, there are more and more observations suggesting that clonal expansion of EBV-infected T or NK cells could be associated with CAEBV infection.^59^,^42^,^60^,^47^,^61^,^56^,^62^,^53^,^54^

Recently, a pediatric survey of CAEBV has been described also in China^63^, even though the death rate was less significant (26,2%) than that reported in Japan (43–61,5%).

A review of the criteria for CAEBV infection appears to be necessary, based on these recents findings. Okano et al.^52^ started this review. This Author, even proposing again the definition of “CAEBV infection”, with some variations previously advanced by himself,^41^ highlights the persistent confusion in making such diagnosis, particularly of the more severe forms because of verying clinical manifestations, outcomes, and association with certain underlying diseases, mainly LPD derived from T cell or NK-cell lineages.

So, he proposed the diagnostic criteria by which underlying diseases should be accurately diagnosed, and when the associated disease is defined, the name of each disease should be used rather than “CAEBV”. The latter term should refer only to cases without an underlying disease at the time of diagnosis.

This type of review inevitably leads to a reduction of chronic forms of EBV infection, most of which could be considered “chronic EBV-associated lymphoproliferative disorders” rather than “chronic EBV infections”. Anyway, their study first of all demonstrated that these forms are characterized by a clonal expansion of T or NK cells infected by EBV. Secondly, it was shown that this result is made possible by the fact that EBV-infected T and NK cells express only EB nuclear antigen 1 and latent membrane protein 1 (latent infection type 2). Since these two proteins are less antigenic, infected cells can escape the immunosurveillance by cytotoxic T lymphocytes. Therefore they may proliferate and cause chronic infection.^49^

There are several noteworthy differences between the T- and NK-cell types of CAEBV infection. T-cell type of the disease is characterized by fever and high titers of EBV-related antibodies, whereas hypersensitivity to mosquito bites and high titers of IgE are observed in patients with the NK-cell type of CAEBV infection.^49^ Cell type infection is important also in predicting the related prognosis, because the survival rates are different between the two groups: T-cells type of CAEBV infection has shorter survival times than those with NK-cell type of infection.^49^

In healthy carriers of EBV, the virus exists latently in resting memory B cell.^13^ It is unclear whether the invasion of blood cells others that B cell causes CAEBV infection or the invasion is an ordinary event, but the host’s immunologic abnormalities allow the expansion of these cells. Answering these questions may have important consequences on our knowledge in relation to other forms of chronic EBV infection which are not presently known in their exact entity and frequency.

There is no doubt that the possibility to search for EBV-DNA in PBMCs, plasma, and especially in tissues should allow to a better investigation of the still confused diagnostic process of the so-called “chronic mononucleosis”. Surely the latest findings on CAEBV have reduced the diagnostic value of the high titer of antibodies, while have increased the role of an high EBV load in PBMCs of these patients (> 2.5 copies / μg EBVDNA) in terms of diagnosis, prognosis and response to therapy.^64^

Therefore, the simple presence of EBV-DNA in plasma may have also the significance of a CAEBV infection since plasma of healthy subjects does not usually contain EBV-DNA.

### b) Chronic fatigue syndrome (CFS):

Chronic fatigue syndrome (CFS) is a new name for a centuries’old disorder characterized by chronic fatigue and multiple somatic symptoms. Syndromes resembling CFS in the eighteenth, nineteenth, and early twentieth centuries were known as febricula, neurasthenia and Da Costa’s syndrome, respectively.^65^ In the 1920s through 1950s, many such cases were attributed to chronic brucellosis.^66^ As mentiones, in the mid-1980s, at least four reports described a syndrome consisting of fatigue, multiple somatic complaints, and elevated antibody titers to Epstein–Barr viral antigens 30, 31, 32, 33. Many chronically fatigued patients were subsequently diagnosed as having ”*chronic EBV disease”, ”chronic EBV syndrome”*, or *“chronic mononucleosis”*.^35^

In 1987 the Division of Viral Diseases of the CDC (Atlanta, USA) called a meeting for investigators and clinicians to develop a consensus on the features of the chronic EBV syndrome. The group doubted that EBV infection and the symptom complex known as “chronic EBV syndrome” were causally related. Therefore chronic EBV syndrome was renamed as *“chronic fatigue syndrome”* and a case definition of this syndrome was devised (**Table 6**).^67^

At a workshop of the National Institute of Allergy and Infectious Diseases (NIAID) in 1991, the CFS case definition was modified by excluding specific psychiatric diagnoses and types of post-infectious fatigue and including selected confounding diagnoses that overlap with CFS (**Table 7**).

In 1994 the International CFS Study Group, coordinated by the American CDC,^68^ proposed a further revision of the previous CFS case definition of 1988, receiving notable. According to the latter case definition, the still more accepted, CFS is defined as a syndrome characterized by the presence, for at least six months, of a severe fatigue, persistent and/or recurrent, with recent or well defined onset, not derived from excessive physical activity, not relieved with rest and that results in a substantial reduction in previous levels of daily activities (school, work, social or personal). To confirm such diagnosis, there must be associated at least four of the following symptoms, not existing prior to the onset of fatigue and persistent or recurrent for six months or more:

1. short-term memory and/or concentration impairment;
2. sore throat;
3. tenderness of the cervical and/or axillary lymph nodes;
4. myalgias;
5. polyarthralgias (without any sign of inflammation);
6. headache with characteristics previously unusual;
7. unrefreshed sleep;
8. malaise for more than 24 hours after exercise.

**Figure 1.**
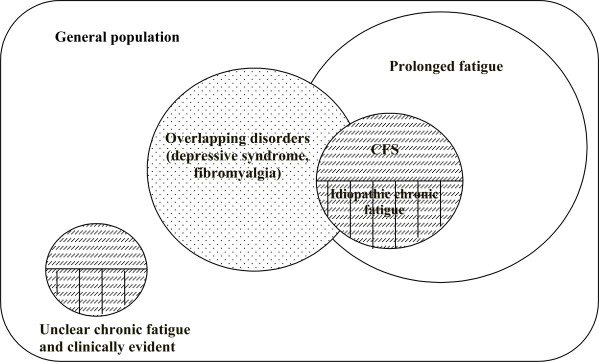
Classification of fatigue (Fukuda et al 68).

One of the merits of the systematization proposed by CDC in 1994 was to have placed the CFS among other forms of chronic or prolonged fatigue, where a diagnosis of CFS is still compatible with some of them when there is an overlapping (e.g.: fibromyalgia syndrome and reactive depression) (**Figure 1**), while CFS must be excluded if others forms are present as established by the exclusion criteria listed in the **table 7**. Moreover, the revised case definition of 1994 allowed the collection of more reliable epidemiological data: the prevalence of CFS varies from 2.3 to 7.4 per 100,000 persons > 18 years of age in 4 surveillance studies in the USA;^69^ it climbs to 560 cases per 100,000 in Britain^70^ and finally to 37 and 127 cases respectively in Australia and New Zealand.^71^

The most affected age is between 20 and 40 years, the female is represented as a ratio F/M = 2–3/1.

The diagnosis of CFS is primarily a clinical diagnosis, based on symptoms as listed in the most recent case definition. The severity of the disease may vary from subject to subject and the more severe stages are usually observed in early phases of the illness.^72^ The course is characterized by alternating phases of remission and reactivation.^72^,^73^,^74^ The prognosis remains favourable, unlikely the disease is progressive, particularly in those forms characterized by an acute onset or with a less presence of psychiatric symptoms.^74^,^75^ To date, the natural history of the syndrome is still poor unknown, neither it is possible to predict its clinical course, nor to identify any specific risk factors associated with it, such as an increased risk of malignancies.^75^, ^77^

Since the diagnosis of CFS is yet a diagnosis of exclusion, it is worth to underline the importance of the exclusion of a primary depression. In particular, it is important that primary depression must be distinguished from reactive and not psychogenic depressive syndromes, often associated with CFS, and considered compatible with CFS diagnosis. Even somatoform disorders do not exclude the diagnosis of CFS, although the still insufficient knowledge about the neurobiological bases of fatigue have often led in the past to consider the syndrome in the same way as a somatization. It was discussed at length, in fact, if fatigue must be numbered among the somatoform disorders, which are also much more frequent in patients who meet the case definition of CFS compared to other clinical conditions characterized by fatigue. There is no doubt that the diagnostic misclassification with primary depression and somatoform disorders accounted for and is the main obstacle to overcome to ensure the specificity of the syndrome^78^. It will not be probably resolved until it will be more light on the pathogenetic mechanism/s of fatigue that occurs in many organic diseases, but also in psychiatric syndromes such as primary depression or in the CFS as currently defined. For this reason the following CFS case definitions, such as the recent Canadian one,^79^ on the basis of clinical experiences with very large numbers of patients, are still hardly employed in clinical and research practice (see **Table 8**).^80^

The studies so far carried out, including the our ones conducted at the Reference Center for CFS at the Clinic of Infectious Diseases of Chieti University (Italy), suggest that CFS is a heterogeneous, probably multifactorial disease.^81^,^82^,^83^,^84^,^85^,^86^ It could also include different diseases from an etio-pathogenetic point of view but showing the same symptoms.

**Figure 2.**
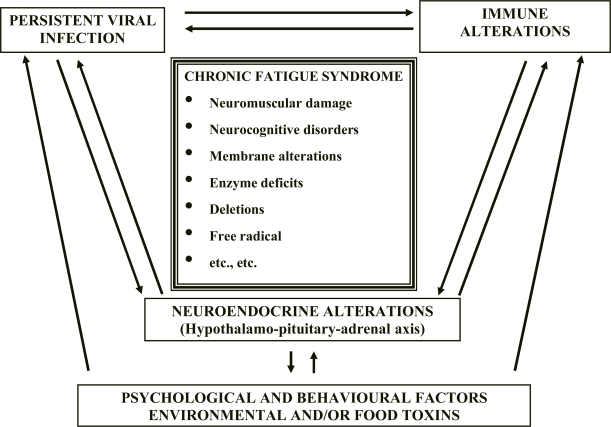
CFS: Pathogenesis

The most accepted pathogenic hypothesis suggests the intervention in the syndrome onset of different factors that can interact with each other, although not all always present in the same patient.^82^,^83^,^85^,^86^ They are represented by latent and/or chronic infections, immune and/or neuroendocrine dysfunctions,^87^,^88^,^89^ environmental and/or food toxins,^90^,^91^,^92^ psychological and behavioural factors (**Figure 2**).^93^,^76^

Among the triggering events in individuals showing a probable genetic predisposition, the infectious agents, particularly the viral ones, seem to play a crucial role, even because their persistence could be also responsible for the immune alterations reported in CFS as well.^87^,^88^ Then, the immune alterations should facilitate the reactivation of latent infections, so to maintain in turn a vicious circle that, through a chronic activation of the immune system, is one of the most accredited pathogenetic substrates for the persistence of a condition of chronic fatigue and/or related symptoms.^82^

The infectious (or post-viral) pathogenic hypothesis is justified not only by the description of outbreaks caused by viral agents, 94,95,96,97 but also by the relative frequency in CFS patients of infectious symptoms at the onset of the disease. In some cases they are well characterized infections (chickenpox, rubella, infectious mononucleosis or other herpesvirus infections from CMV, HHV-6, etc.); more often the symptomatology is nonspecific, flu-like type, with sore throat, fever, muscle aches, diarrhea, etc.^35^,^3^,^72^

Among the potential viruses involved in CFS onset, EBV is primarily because, as mentioned above, the syndrome was initially described in the ‘80s in patients with serological signs of a chronic or persistent EBV infection.^30^,^32^,^33^ However, even in the so-called “Lake Tahoe” outbreak occurred in Nevada (USA),^98^,^35^ the American epidemiologists have been unable to exclude the possibility that high levels of anti-EBV antibodies in CFS cases, on which the causative correlation was based, could represent only the epiphenomenon of a polyclonal activation of B lymphocytes, as demonstrated by the concomitant presence of higher antibody levels compared with controls, even against other viruses (CMV, HSV1 and 2, measles virus).^35^,^15^

Another potential virus related to CFS onset is HHV-6, described for the first time right at the time of the Lake Tahoe outbreak, and immediately suspected of a possible link with the epidemic cluster^99^ and then with CFS. In particular, in respect of a possible etiopathogenetic link between HHV-6 and CFS onset, many studies were subsequently report.^100^ Chapenko et al.^101^ recently found a significantly higher prevalence of persistent/latent HHV6 infections and dual HHV6 and HHV7 infections among patients with CFS.

In a recent study Hickie et al.^102^ demonstrated that CFS is a fairly common sequel of several types of viral and non viral infections including EBV, Q fever and Ross River virus. This work confirms previous findings that the severity of the acute illness rather than the infective pathogen appeared to be the critical determinant of post-infective fatigue syndrome.^103^

Similar evidences seem to support a causal role of other microrganisms in CFS pathogenesis, such as enteroviruses^104^,^105^,^106^ and gram-negative enterobacteria.^107^ In addition, more recently the paper of Lombardi et al.^108^ published on *Science* journal has excited a big sensation since it showed in 67% of CFS examined patients the presence of a retrovirus related to, but different from, known xenotropic murine leukemia virus (XMRV). However, since at least other three studies did not support this finding,^109^,^110^,^111^ the following hypotheses have been advanced:

1. the distribution of XMRV could be much lower in Europe than in USA and CFS may have varied environmental triggers in different parts of the world;
2. more different sequences of XMRV could be present than previously reported and the primer sequences employed in different studies could be not exact;
3. the replication rate for XMRV could be very low and/or the levels could fluctuate over time in a given individual; in such cases a single round PCR of genomic DNA isolated from PBMCs might not result in the amplification of XMRV, even from an infected individual.

Independently from the inclusion of this new virus in the very numerous directory of infectious agents potentially implied in CFS pathogenesis, in the clinical history of some CFS patients the timeline between the infection and the onset of the syndrome is so obvious and well documented that it is hard to deny at the first such a role in triggering the second. This is particularly true for the infections caused by the herpesviruses (mainly by EBV), which seems to have an important pathogenetic role for at least a substantial subset of patients with CFS.

Independently of a role of viral infection in CFS onset or during the course of the same, the assumption on which most are working is that the pathogenic substrate of fatigue may be the same.

A growing role has been attributed, by some years to now, to the oxidative damage which, according to the Pall’s hypotheses,^112^ derives from the release of pro-inflammatory cytokines induced by the trigger factor (viral infection and not only). The cytokines would activate the inducible form of nitric oxide synthase (i NOS) and then stimulate the production of nitric oxide which, in turn, would interact with other reactive substances, such as superoxide anion, to lead to the production of a highly reactive compound as peroxynitrite (OONO). A reduced oxidative metabolism with an increased production of lactic acid had been previously shown by our group^113^ in skeletal muscle of CFS patients. In addition, Australian researchers^114^ found higher blood levels of markers of oxidative damage in patients with CFS, well related to the clinical expression of CFS symptoms. A significant increase in circulating levels of markers of oxidative damage (T-BARS) and an equally significant reduction in the duration of lag-phase and vitamin E levels were also found by our group^115^ both in plasma and in LDL in patients with CFS compared with controls. The potential consequences of these observations in a therapeutic way are obvious as, for example, the administration of vitamin E would be able to correct the so observed blood alterations. The clinical trial that we are performing will allow us to assess whether both the oxidative stress can be resolved at neuromuscular level by this or other anti-oxidants, and the alteration of membrane lipids can be modified, for example by a supplementation with unsatured fatty acids, which also were effective in the only controlled trial so far conducted on CFS^116^.

The purposes of this review on the potential role of EBV in CFS onset do not include the analysis of the new studies that, in the past few years, have confirmed the role of the oxidative damage potentially triggered from the infectious agent^114^,^117^,^118^,^119^,^120^,^121^ and of the activation of the immune system directly induced by the latent infection or the oxidative stress.^122^ New horizons in the pathogenesis of CFS come from the genomic and the proteomic that showed: a dysregulation of mithocondrial and ion channel regulatory genes, upregulation of proinflammatory cytokine pathways and different patterns of endocrine, immune and metabolic dysregulation that identified as many six subgroups of CFS. Although the investigators could not identify a definitive genetic markers for CFS, they were able to identify 28 single nucleotide polymorphism to predict with 76% accuracy whether an individual had CFS^122^. In particular, a study by Vernon et al.^123^ described alterations in gene transcripts among patients with post-infective fatigue following acute infection with EBV. They found that the patients which developed the post-infective fatigue syndrome had gene expression profiles that indicated an altered host response during acute episode of IM when compared with those patients who have recovered without event.

In conclusion, although there are no compelling data to suggest that the CFS commonly results from chronic infection, it is tempting to consider that a subset of patients possesses mild, chronic EBV infection, primarily patients in whom the illness clearly began with a primary EBV infection or those in whom antibodies to EBNA proteins are deficient.

Although numerous observational data support this thesis since a chronic reactivation of latent infections has been often documented in CFS patients by determining with the PCR technique viral nucleic acids both in blood and tissues, particularly of EBV, it is more appropriate and satisfying, however, to speculate that the syndrome represents a general response to a variety of psychological, physical, chemical, immunological and virological irritants.

The experience of other systemic and nonspecific effects of the body’s response to acute or chronic reactivated infections, and in particular the release of potent lymphokines and mediators of inflammation, may be considered the piece that unifies the various pathogenic theories, along with oxidative damage which could be a legitimate consequence of them at tissue level.

Even if the pathogenic role of a chronic EBV infection in a number of CFS patients is probable, there is no doubt that it should be distinguished from much more rare CAEBV cases, and in particular the more severe ones, because of significant differences in symptomatic and prognostic type.

## Chronic Hepatitis EBV-Related

A liver involvement by EBV is always present in the acute onset of infection (IM) and is characterized by a transient, self-limited elevation of liver enzymes.^124^ In about 5–7% of cases jaundice may be the presenting symptom together with a prominent cholestatic picture.^125^,^126^ Severe hepatitis is infrequent at the time of primary EBV infection of immunocompetent subjects.^127^,^128^

Some cases of autoimmune^129^,^130^ or granulomatous^131^ hepatitis have also been associated with EBV infection.

Liver damage during the acute EBV infection is hard to understand since the virus does not seem to be able to infect hepatocytes.^132^ These cells could be affected indirectly by mediators that are released in the liver as a consequence of the cytotoxic T lymphocyte attack against B lymphocytes infiltrated in the liver parenchyma and infected by the virus.^132^ If this or another mechanism of hepatotoxicity can be present also in the chronic and/or latent EBV infection following the acute phase, supporting a possible EBV chronic liver disease, remains a problem not yet well defined, due to the rarity of reports of EBV-related chronic hepatitis, described at most among the clinical manifestations of CAEBV and in particular of severe CAEBV.^133^,^134^

Through the evaluation of liver biopsies of 21 patients who were positive for EBV-DNA with *in situ* hybridization, Drebber et al.^135^ made a diagnosis of acute hepatitis in 10 cases, of subacute hepatitis in 3 and chronic hepatitis in 7; in one case there was a chronic cholangitis at the histologic examination. At liver biopsies evaluation of patients with “cryptogenic chronic hepatitis” the search for EBV-DNA was positive in 7 out of 68 (10,3%). In these cases, however, it remains unclear whether EBV-DNA positivity in the liver was suggestive for an etiopathogenetic relationship, or simply an epiphenomenon of possible reactivation of latent EBV infection. It is noteworthy, however, that the same AA. did not found any presence of EBV-DNA in liver biopsies of 20 patients with liver steatosis.

In recent literature always limited reports of EBV-related chronic hepatitis appeared.^136^ These Authors argue that chronic liver disease may be the only clinical manifestation of chronic EBV infection when characterized by persistent low viral replication or by frequent reactivations. Liver damage seems to be indirect, possibly linked to the action of cytotoxic TCD8+ lymphocytes specifically activated by EBV and of the cytokines induced by such activation.

If an albeit limited etiopathogenetic role in cryptogenic chronic liver disease seems to be recognized for EBV, this remains difficult to be demonstrated with the current available techniques. Furthermore, the possible role of EBV in hepatocellular carcinogenesis (HCC) is even more uncertain, although EBV-DNA was detected in samples of HCC with the techniques of molecular biology, often along with DNA of other oncogenic viruses (HBV and HCV).^137^ A co-carcinogenic role also for hepatic tumors can not be yet excluded.

## Neurological EBV infections

In a surveillance study performed in Japan from 1989 to 1991, the incidence of EBV encephalitis was 0.5 cases per million persons per year.^138^ So, EBV-associated neurological syndromes represent rare complications in infectious mononucleosis, and include acute encephalitis, meningitis, acute disseminated encephalomyelitis (ADEM), cerebellitis and myelitis.

Within these complications, prolonged or relapsing forms have been described and, in according to specific serological tests for EBV, they seem to be associated with chronic EBV infection.^139^,^140^,^141^ These neurological complications seem to well respond to steroid therapy and thus they must be considered of a post-infective type.

Since many reports of neurological complications during chronic EBV infections characterized by a prolonged or relapsing course come mainly from Japan, it seems that they must be included among clinical pictures of CAEBV, and particularly of the severe forms.

However, since EBV certainly shows a good neurotropism, EBV-DNA must be looked for both in cerebrospinal fluid and tissues in all neurological forms of unknown etiology, especially in ADEM and in those forms characterized by a chronic and/or relapsing course. The same EBV neurotropism could be involved both in outbreaks of myalgic encephalomyelitis, so described before that the new case definition of “chronic fatigue syndrome” would allow a more precise framework, and in cases of CFS that show more similarities in a clinical point of view with infectious and especially post-infective diseases of CNS.

## Conclusions

In conclusion, the dilemma of clinical forms of chronic EBV infection is far from be resolved.

Since there is a wide variety of clinical syndromes with which the persistent and/or chronic and/or latent EBV infection has been linked, it is extremely difficult to identify a univocal pathogenetic link.

Furthermore, the well studied and documented correlation between this virus and oncogenetic events certainly does not facilitate the task. Hence the need to distinguish these events from those that are implied with organ manifestations, neuroendocrine syndromes and/or post-infective sense. For the latter, like the oncogenetic events, it is likely that a crucial role is played by predisposing characteristics - such as “genetic” and/or “environmental” ones – of the host, although a causal role of eventual EBV mutant strains can not be excluded.

Since the EBV infection is a latent infection characterized by more or less frequent reactivations, which are in turn conditioned by genetic and environmental factors, an important role should be played by the very different latency programs, that have been widely studied in the context of EBV-related cancer events and that would deserve to be examined thoroughly in their aspects and their consequences, even in the chronic complications of EBV infection or presumed such.

It is not a case that genomic and proteomic in the past few years represent the field of productive studies in the within of the research on “chronic fatigue syndrome” and on its relationships with the chronic infection from EBV or from other causal agents of latent or persistent infections, viral and not only viral.^142^,^143^,^144^,^145^,^146^,^123^,^147^,^148^,^149^,^150^

## Figures and Tables

**Table 1.** EBV latent viral genes (from Crawford DH^7^).

***GENE***	**GENE PRODUCT**	**POSTULATED FUNCTION**
*BKRF1*	EBNA-1	viral genome maintenance essential for *in vitro* immortalization of B cells

*BYRF1*	EBNA-2	viral oncogene essential for *in vitro* B-cells immortalization

*BLF3-BERF1*	EBNA-3A	essential for in vitro B-cell immortalization
*BERF2α-BERF2β*	EBNA-3B	not known
*BERF3-BERF4*	EBNA-3C	viral oncogene essential for in vitro B-cell immortalization

*BWRF1*	EBNA-LP	not known
*BNLF1*	LMP-1	induces lymphoblastoid phenotype tumor necrosis factor receptor superfamily member

*BARF1/BNRF1*	LMP-2A	prevents cell activation and lytic-cycle entry
*BNRF1*	LMP-2B	not known
*BCRF1*	EBER1, EBER2	regulates PKC activity upregulates *bcl2* on Burkitt’s lymphoma cells
*BARF0*	BamHIA transcripts	not known

Abbreviations: EBNA: Epstein-Barr viral nuclear antigen; LMP-1: Latent membrane protein; EBER: Epstein-Barr viral small RNA; PKC: Protein kinase C.

**Table 2.** Expression of EBV latent genes in disease (from Cohen 13)*.

**PATTERN OF LATENCY**	**EBNA-1**	**EBNA-2**	**EBNA-3**	**LMP-1**	**LMP-2**	**EBER**	**DISEASE**
Type 1	+	−	−	−	−	+	Burkitt’s lymphoma

Type 2	+	−	−	+	+	+	Nasopharyngeal carcinoma, Hodgkin’s disease, peripheral T-cell lymphoma

Type 3	+	+	+	+	+	+	Lymphoproliferative disease, X-linked lymphoproliferative disease Infectious mononucleosis

Other	±	−	−	−	+	+	Healthy carrier

Abbreviations: EBNA: Epstein-Barr viral nuclear antigen; LMP: Latent membrane protein; EBER: Epstein-Barr virus encoder RNA.

* A plus sign indicates that the gene is expressed in the disease, a minus sign that is not expressed, and the two together that the gene may or not be expressed

**Table 3.** EBV chronic infection and associated clinical syndromes (adapted from Okano 41).

**CATEGORY**	**CHARACTERISTICS OF:**		
	**CFS**	**CAEBV**	**SCAEBV**
**Major clinical manifestation**	Often debilitating fatigue and fever	Fever, lymphoadenopathy and fatigue (onset begins with acute IM)	See CAEBV + hepatosplenomegaly (severe) and a tendency for pancytopenia
**Age distribution**	Mostly adults	Mostly adults	Mostly children (< 15 years)
**Antibody titers to EBV**	Normal, seropositive or seronegative	Reactivation with moderately high antibody titers of VCA IgG and EA IgG and with low antibody titers to EBNA	Extremely high antibody titers of VCA IgG (> 5,120) and EA (≥ 640) and positivity for VCA IgA and EA IgA
**EBV-DNA with PCR**	±	+ (high viral load)	++ (very high viral load)
**Other designations or acronyms in the literature**	Chronic symptomatic EBV infection, chronic mononucleosis, chronic acitve EBV infection (CAEBV), chronic fatigue and immune dysfunction syndrome (CFIDS)	CFS, chronic symptomatic EBV infection, CAEBV, chronic mononucleosis	CAEBV

Abbreviations: CFS: chronic fatigue syndrome; CEBV: chronic active EBV infection; SCAEBV: severe chronic active EBV infection.

**Table 4.** Proposed guidelines for diagnosing CAEBV* (from Okano 52).

Persistent or recurrent IM-like symptom Unusual pattern of anti-EBV antibodies with raised anti-VCA and anti-EA, and/or detection of increased EBV genomes in affected tissues, including the peripheral blood Chronic illness which cannot be explained by other known disease processes at diagnosis †

Abbreviations: CAEBV: chronic active Epstein-Barr virus infection; IM: infectious mononucleosis; VCA: viral capsid antigen; EA: early antigen; LPD: lymphoproliferative disorder.

* A case of CAEBV must fulfill each category.

† An EBV- associated disease such as hemophagocitic lymphohistiocytosis or LPD/lymphoma mainly derived from T-cell or NK-cell lineage often develops during the course of illness; some patients also suffer from cutaneous lesions, such as hypersensitivity to mosquito bites.

**Table 5.** Supplemental findings and recommended specific laboratory tests for diagnosing CAEBV (from Okano 52).

IM-like symptoms generally include fever, swelling of lymph nodes, and hepatosplenomegaly; additional complications include hematological, digestive tract, neurological, pulmonary, ocular, dermal and/or cardiovascular disorders (including aneurysm and valvular disease) that mostly have been reported in patients with IM Anti-EBV antibodies with raised anti-VCA and anti-EA ordinarily consist of VCA-IgG ≥ 1:640 and EA-IgG ≥ 1:160; positive IgA antibodies to VCA and/or EA are often demonstrated Recommended specific laboratory tests Detection of EBV-DNA, RNA, related antigens and clonality in affected tissue including the peripheral blood PCR (quantitative, qualitative) More than 10 2,5 copies /μg DNA are generally detected in peripheral blood mononuclear cells; healthy individuals occasionally show positive results by qualitative PCR analysis In situ hybridization (e.g., EBERs) Immunofluorescence etc. (e.g., EBNA, LMP) Southern blotting (including clonality of EBV) Clarifyng a target cells of EBV infection Double staining of EBNA or detection of EBER or EBV DNA with each marker for B, T, NK, cells or monocytes/ macrophage/ histiocytes is recommended by using such methods as immunofluorescence, immunohistological staining, or magnetic beads Histopatological and molecular evaluation General histopathology Immunohistological staining Chromosomal analysis Rearrangement studies (e.g., immunoglobulin, T-cell receptor) Immunological studies Generalized immunological studies Marker analysis of peripheral blood (including HLA-DR) Cytokine analysis

Abbreviations: IM: infectious mononucleosis; VCA: viral capsid antigen; EA: early antigen; PCR: polymerase chain reaction; EBERs: EBV-encoded RNAs; EBNA: EBV-determined nuclear antigen; LMP: latent membrane protein; HLA: human leukocyte antigen.

**Table 6.** Centers for Disease Control’s case definition of the chronic fatigue syndrome (CFS) (from Klonoff 73)*.

**Major Criteria** New onset of fatigue lasting 6 months reducing activity to < 50% Other conditions producing fatigue must be ruled out
**Minor Criteria**
*Symptom Criteria* – beginning at or after onset of fatigue and persisting or recurring for at least 6 months Low-grade fever: temperature of 37,5°C–38,6°C (99,5°F–101,5°F) orally or chills Sore throat Painful cervical or axillary limph nodes Generalized muscle weakness Muscle pain Postexertional fatigue lasting 24 hours Headache Migratory arthralgias Nueropsychological complaints (photophobia, transient visual scotoma, forgetfulness, excessive irritabilitity, confusion, difficulty thinking, inability to concentrate or depression) Sleep disturbance Acute onset of symptoms over a few hours to a few days
*Physical Criteria* – documented by a phisician twice, at least 1 month apart Low-grade fever: temperature of 37,6°C–38,6°C (99,7°F–101,5°F) orally or 37,8–38,8°C (100,0°F–101,8°F) rectally Nonexudative pharyngitis Palpable cervical or axillary lymph nodes up to 2 cm in diameter

* A case of CFS must fulfill both major criteria as well as reither eight symptom criteria or six symptom criteria plus two physical criteria.

**Table 7.** National Institute of Allergy and Infectious Diseases/National Institute of Mental Health’s modifications of the Centers for Disease Control’s case definition of the chronic fatigue syndrome (from Klonoff 73).

**Exclusion** Psychiatric disorders Psychoses Psychotic depression Bipolar disorder Schizophrenia Substance abuse Postinfectious fatigue (must include A, B and C) Establishment of a definite etiology An etiologic agent known to regularly produce chronic active infection A clinical picture compatible with ongoing active infection Chronic hepatitis B or C with active liver disease Infection with human immunodeficiency virus Lyme disease (inadequately treated) Tuberculosis
**Inclusion** (in patients who otherwise meet the Centers for Disease Control’case definition) Fibromyalgia Postinfectious fatigue Lyme disease with persistent fatigue after appropriate antibiotic therapy Brucellosis with persistent fatigue after appropriate antibiotic therapy Acute infectious mononucleosis (documented) followed by chronic debilitating fatigue Acute cytomegalovirus infection Acute toxoplasmosis (adequately treated) Nonpsycotic depression Somatoform disorders Generalized anxiety disorder/panic disorder

**Table 8.** Diagnostic criteria (adults) for CFS-like illness 1988–2003 (from Vance 80).

- US Centers for Disease Control and Prevention (CFS – Holmes et al, 1988). - London (ME – Dowsett et al, 1990). - Australia (CFS- Lloyd et al, 1990). - World Health Organization, 1994 (non clinical). - US Centers for Disease Control and Prevention (CFS – Fukuda et al, 1994). - “Canadian” expert consensus clinical case definition for ME/CFS (Carruthers et al, 2003).
**Previous literature:** - Epidemic neuromyasthenia (Parish et al, 1978) - Myalgic encephalomyelitis (Acheson, 1959) - Epidemic neuromyastenia (Henderson and Shelokov, 1959)
